# Dyadic Conversation between Mandarin-Chinese-Speaking Healthy Older Adults: From Analyses of Conversation Turns and Speaking Roles

**DOI:** 10.3390/bs13020134

**Published:** 2023-02-05

**Authors:** Meng-Ju Tsai

**Affiliations:** 1 Department of Speech-Language Pathology and Audiology, Chung Shan Medical University, Taichung City 402, Taiwan; mjtsai@csmu.edu.tw; 2 Speech and Language Therapy Room, Chung Shan Medical University Hospital, Taichung City 402, Taiwan

**Keywords:** conversation, Mandarin Chinese, older adults, conversation turns, speaking roles, health care

## Abstract

Older adults’ daily conversations with other older adults enable them to connect to their surrounding communities and improve their friendships. However, typical aging processes and fluctuations in family caring might cause conversation changes. The purpose of this study was to explore the quantitative contributions of conversation turns (CTs) and speaking roles (SRs) in Mandarin-Chinese-speaking conversation dyads between mutually familiar healthy older adults (HOAs). A total of 20 HOAs aged 65 or over were recruited. Each dyad conversed for ten minutes once a week for five weeks, five sessions per dyad, for a total of 50 sessions. The frequency and percentages of the coded CTs and SRs contributed by each HOA were individually tallied and calculated. Quantitatively symmetrical contributions of CTs and SRs occurred in Mandarin-Chinese-speaking conversation dyads between mutually familiar HOAs. Although typical aging processes might change conversations, both Mandarin-Chinese-speaking HOAs serve as active interlocutors to each other in taking CTs and SRs to co-construct their conversation processes and content in their dyadic conversation. Sufficient knowledge of conversation co-constructions might lead them to have more supportive environments to connect to surrounding communities and improve their friendships.

## 1. Introduction

### 1.1. Older Adults in Taiwan

In Taiwan, the *Senior Citizens Welfare Act*, released in 2015 by the Ministry of Health and Welfare [1], defines older adults as people aged 65 years and above. In 2022, the population of older adults in Taiwan was 17.6 %, approximately 3.98 million, and this will increase to 20.0% in 2025, making Taiwan a super-aged society [2]. As the population age increases, addressing the needs of older adults will become increasingly important [3]. Moreover, young-old adults (aged 65–74) giving care to old-old adults (aged over 75) has gradually become a common phenomenon [4], and maintaining the mental health of older adults and keeping them active is one of the most demanding issues in social welfare [3]. This is both a developing [5] and challenging task [6]. In response to this growing problem, community care centers for older adults were established to provide primary prevention care services, including caring visits, telephone consultations, referral services, catering services, and health promotion activities (e.g., physical activities), to increase their life quality and community participation [7,8]. The number of people served by community care centers in Taiwan from 2014 to 2021 increased by 55.6% [9]. Daily conversations among these older adults in the community care centers enable them to connect to the surrounding community (i.e., caregivers and neighbors) and improve their friendships [10,11,12,13,14]. Accordingly, maintaining their identity; relieving loneliness; reducing depression or anxiety [12,15]; increasing self-esteem, well-being, and social relationships; and improving contributions to the community, positive attitudes toward aging processes, and understanding of the typical aging process [12,16,17] can be anticipated from their conversations.

However, typical aging processes (e.g., declined cognition and physiologic changes in hearing, voice, and speech processes) and fluctuating external environments (e.g., family caring) might cause conversation changes [17,18,19]. Some healthy older adults (HOAs) try to prevent these changes, and some HOAs struggle to maintain the current conversation thread [12]. These changes frequently include disturbances of speech (e.g., unintelligible speech), declined language skills (e.g., declined comprehension of complex utterances and naming), and difficulty participating in conversations [12,20]. Li [21] emphasized that support to HOAs from family members (e.g., caregivers) is critical. Although HOAs might actively, rather than passively, participate in their dyadic conversations [22], their caregivers (e.g., young-old adults aged 65–74) also have substantial roles in listening, reflecting, and offering advice [12,23].

### 1.2. Dyadic Conversation

A dyadic conversation is defined as two interlocutors participating in a conversation and involves collaboration influenced by a speaking interlocutor, a listening interlocutor, and conversation behaviors (i.e., verbal and nonverbal conversation behaviors) [24,25,26,27]. The speaking and listening interlocutors take turns using recognizable conversation behaviors to express and comprehend messages during their dyadic conversation [28]. A successful dyadic conversation requires co-constructions [25,26], which are social processes operated by two or more interlocutors [29]. Positioning theory characterizes these interlocutors subjectively, and the interlocutors jointly co-construct their conversation [30], by which they dynamically alter their conversation behaviors with respect to the ongoing and anticipated behaviors of the other interlocutor [31]. These dynamic alternations are influenced by the interlocutors’ shared personal experiences and shared world knowledge [32]. Generally, conversation processes (e.g., completing incomplete conversation turns) and conversation content (e.g., building mutual meanings) are co-constructed [25] and influence and are influenced by other interlocutors rather than by the modes of conversation behaviors (e.g., verbal and nonverbal conversation behaviors) [33]. Through the co-constructions, the occurrence of conversation breakdowns can be minimized [26]. However, these co-constructions cannot be observed on cognition tests [34]. It is known that the co-constructions of the conversation process can be explored based on conversation turns (CTs) [35] and that the co-constructions of conversation content can be explored based on speaking roles (SRs) [36,37].

CTs are dominant elements of conversation across all modes of conversation behaviors (e.g., verbal and nonverbal conversation behaviors) [38] and critically contribute to conversations [39]. Healthy interlocutors across different languages and cultures usually take several CTs to exchange thoughts without difficulties and construct their conversation sequentially [40]. CTs have a variety of definitions; for example, a conversation occurring when speaking interlocutors change. When one speaking interlocutor says “hello,” and a listening interlocutor also says “hi,” a CT is separately contributed by the speaking interlocutor and listening interlocutor [41]. Norén et al. [42] argued that the number of CTs could be analyzed to report the two interlocutors’ contributions within their dyadic conversation.

In 1981, Goffman [43] proposed a concept of SRs, including animators, authors, and principals. An animator is an interlocutor who produces utterances, an author is an interlocutor who selects the sentiments and words being expressed, and a principal is an interlocutor whose position and beliefs are established by the words spoken [43]. Extract 1 illustrates these three roles. In line 1, A verbally asks a question, simultaneously contributing as the animator, author, and principal. In line 2, B verbally answers A’s question, contributing as the animator, author, and principal. In line 3, A restates B’s role, contributing as the animator and author, while B contributes as the principal. That is, the animator and author roles were played by A, but the principal was played by B in line 3.

Extract 1

1. A: 你有跟家人住嗎 (/nǐ yǒu gēn jia rén jhù ma˚/, do you live with your family?)
2. B: 有和太太 (/yǒu hé tài tai˚/, yes with my wife)
3. A: 喔跟太太哦 (/wò gēn tài tai˚é/, oh, with your wife)

Conversation content is used to determine the contributions of these three roles (i.e., animator, author, and principal) from the interlocutors [39,43,44,45]. Each interlocutor might take one or more than one role of the SRs to co-construct mutual meanings of their conversation content with sharing responsibility [36,37,44]. Tsai et al. [46] emphasized that the analysis of SRs provides the value of highlighting who speaks the messages, who selects the messages, and whose position is spoken in a dyadic conversation.

### 1.3. Mandarin-Chinese-Speaking Conversation

Approximately 66.3% of Taiwanese people over six years of age speak Mandarin Chinese [47], and Mandarin Chinese is the official and dominant language [48,49] of Taiwan. Wu [50] emphasized that modesty, one of the core values of Chinese culture, guides Mandarin Chinese conversation in Chinese culture. For example, healthy interlocutors with Chinese culture broadly perceive their familiar others (e.g., immediate family and intimate friends) as themselves and praise themselves (i.e., self-praise) followed by the other interlocutor’s praise about themselves in an immediately prior CT in their dyadic conversation [50]. The listening interlocutor not only listens to the speaking interlocutor but also actively uses verbal (e.g., 我們的電話; /wǒ men˚de˚ diàn huà/, our telephone) and nonverbal (e.g., head nod) conversation behaviors to shape the sequential organizations of CTs and then construct and negotiate their mutual conversation process and content [51,52]. In Mandarin-Chinese-speaking dyadic conversation, the speaking interlocutors respond orderly and not incidentally immediately after the speaking interlocutors’ CTs, without intervening with the CTs [50,52,53]. Equal and symmetrical contributions of CTs might be expected in these Mandarin-Chinese-speaking dyadic conversations, which is an ideal conversation proposed by Sacks, Schegloff, and Jefferson [28] with no interruptions [28,50]. Nonetheless, these equal and symmetrical contributions sometimes might not easily be accomplished and might be disrupted (Li, 2014).

In the past two decades, different perspectives of Mandarin-Chinese-speaking dyadic conversations were investigated, including cooperative and intrusive interruptions of Mandarin-Chinese-speaking and English-speaking healthy young adults [54] and discourse patterns of Mandarin-Chinese-speaking older adults with Alzheimer’s disease (AD) and HOAs [55]. Li [54] investigated the frequencies of cooperative and intrusive interruptions in 20 same-gender and mutually unfamiliar dyads between healthy young adults, including ten Mandarin-Chinese-speaking dyads (mean age: 30) and ten English-speaking dyads (mean age: 28.22). These adults were asked to be video-recorded in a simulation of a doctor–patient interview at the lab, with a mean time of 5 min 16 s for Mandarin-Chinese-speaking dyads and 4 min 48 s for English-speaking dyads. Cooperative interruption is when a listening interlocutor encourages the speaking interlocutor’s CT (i.e., non-obligatory) to agree, assist, and clarify the ongoing conversation process and/or content [54]. This includes showing concurrence, compliance, understanding, or support to the speaking interlocutor; providing the speaking interlocutor with a word, phrase, sentence, or idea; or having the speaking interlocutor clarify or explain the previous messages that are not clearly understood [54].

In contrast, an intrusive interruption is a process wherein a listening interlocutor who disagrees with the speaking interlocutor’s messages intrusively interrupts to take the speaking interlocutor’s CTs (i.e., non-obligatory) to disrupt the conversation process and/or content [54]. These include disagreeing with the speaking interlocutor’s messages; taking the conversation floor to develop the current interview topic; changing the current interview topic; and summarizing the messages away from listening to unwanted messages [54]. These Mandarin-Chinese-speaking dyadic interviews use more cooperative interruptions than intrusive interruptions, and the opposite trend is found among the English-speaking dyadic interviews. Li (2001) explained that Mandarin-Chinese-speaking interlocutors are more “other”-oriented (e.g., loyalty and solidarity) than English-speaking interlocutors in order to help the other interlocutors along, regardless of their roles. These listening interlocutors in Mandarin-Chinese- and English-speaking young adults take the speaking interlocutors’ CTs (i.e., non-obligatory) to cooperatively and intrusively interrupt the speaking interlocutors’ CTs.

Lai [55] investigated discourse patterns of interviews with 40 mutually unfamiliar Mandarin-Chinese-speaking older adults with AD and HOAs (20 older adults with AD and 20 HOAs) in Taiwan. Lai interviewed these older adults with AD and HOAs about their family, life, day, and national holiday spending. Discourse-building features (e.g., clear information units, cohesion, correct pronouns, and appropriate conjunctions) that continue the conversation and discourse-impairing features (e.g., word-finding difficulties, repetitions, and revisions) that impede the conversation were analyzed. These revisions are used by the interlocutors to achieve the appropriateness and clarity of the messages [55], and these HOAs used more discourse-building features than the older adults with AD in their dyadic interviews.

The early studies in Mandarin-Chinese-speaking interlocutors reported two findings. First, while listening, Mandarin-Chinese-speaking healthy young adults cooperatively and intrusively consider the speaking interlocutors’ CT (i.e., non-obligatory) interruptions in the simulations of doctor–patient interviews [54]. However, symmetries of these cooperative and intrusive interruptions in these dyadic interviews between Mandarin-Chinese-speaking healthy young adults were not addressed. Second, Mandarin-Chinese-speaking HOAs used more discourse-building features than older adults with AD in their dyadic interviews [55]. Symmetries of these discourse-building features and discourse-impairing features in these dyadic interviews with these Mandarin-Chinese-speaking older adults with AD and HOAs were not explored.

Although quick alternations of CTs requiring substantial auditory attention-switching processes might be challenging for HOAs with typical aging processes (e.g., cognitive and sensory decline) in dyadic conversations [20], Carter and Everitt [22] argue that they are active rather than passive interlocutors. Their mutually familiar interlocutors (e.g., friends, family members, and care providers) with shared personal experiences and shared world knowledge serve as important roles in co-constructing their conversation processes (e.g., completing incomplete conversation turns) and conversation content (e.g., building mutual meanings) [12,23]. Given the gradually common contexts in Taiwan of old adults caring for old adults, the occurrence of ideal conversations through equal and symmetrical contributions of CTs proposed by Sacks, Schegloff, and Jefferson [28] in co-constructing conversation processes between mutually familiar Mandarin-Chinese-speaking HOAs is unclear. Furthermore, the quantitative contributions of SRs in co-constructing conversation content among mutually familiar Mandarin-Chinese-speaking HOAs are also unknown.

The current study was part of a large research project to explore the patterns of Mandarin-Chinese-speaking HOAs’ conversations. The study aimed to explore the quantitative contributions of CTs and SRs in Mandarin-Chinese-speaking conversation dyads between mutually familiar HOAs. Two research questions were investigated. First, do quantitatively symmetrical contributions of CTs occur in Mandarin-Chinese-speaking conversation dyads between mutually familiar HOAs? Second, do quantitatively symmetrical contributions of SRs occur in Mandarin-Chinese-speaking conversation dyads between mutually familiar HOAs? In view of these points, this study hypothesizes that the contributions of CTs and SRs in Mandarin-Chinese-speaking conversation dyads between mutually familiar HOAs will be symmetrical.

It has been emphasized that analyzing dyadic conversation is a suitable way to explore aging changes in Mandarin-Chinese-speaking conversation due to its single focus on a minimal set of a speaking interlocutor, a listening interlocutor, and simple alternations of CTs [19,20]. Understanding co-constructions between the interlocutors can be greatly explored in the analysis of dyadic conversations [42]. In addition, quantitative analyses were most frequently used to explore behavioral patterns (e.g., contributions) of dyadic conversation [19,36] and have been used in several previous studies (e.g., [46,56,57]). Individual contributions of each HOA to the dyadic conversation, rather than whole conversation dyads, were recommended to avoid masking each HOA’s differences and demonstrate the effect of diverse characteristics (e.g., aging) [17]. The findings of the current study provide normative information on the quantitative contributions of CTs and SRs in Mandarin-Chinese-speaking conversation dyads between mutually familiar HOAs.

## 2. Methods

### 2.1. Research Participants

Ten dyads (D1 to D10) participated in the study, and each dyad contained two mutually familiar interlocutors, a total of 20 HOAs (ten men and ten women). The first ten HOA men (i.e., core participants) were recruited from local community groups and were requested to recommend one HOA woman (e.g., spouses, siblings, relatives, caregivers, or significant others) from their family and community. They all met the inclusion criteria of (a) being aged 65 or over; (b) using Mandarin Chinese or Taiwanese in their daily conversation environments; (c) having scores of Mini-Mental State Examination (MMSE) over 24 if educated or over 15 if uneducated; (d) hearing between 41–55 dB or better with naked ears or with hearing aids; and (e) being able to participate in basic social conversations. Although the occupation (e.g., more standard language forms for teachers), gender (e.g., expressing more clearly and getting messages across for women), and attributes (e.g., living styles, occupation, education levels, and sensitivity to the conversation) of the HOAs might affect their language use [17], these diversities correspond to heterogeneous groups of HOAs in Taiwan. However, the effect of gender was controlled in the current study. Written informed consent for participating in the study from each HOA was obtained from a legally authorized person [58]. The mean age of the ten core HOAs was 78.5 (71–89), and the mean age of the ten recommended HOAs was 75.7 (68–85). The detailed demographic characteristics are presented in Table 1 and Table 2.

### 2.2. Research Design

A quantitative, non-experimental, descriptive observation was conducted [59], and the quantitative contributions of CTs and SRs in Mandarin-Chinese-speaking conversation dyads between mutually familiar HOAs were explored. Ethics approval was obtained from the Institutional Review Board (IRB). The author from the department of speech–language pathology and audiology and four research assistants were the primary researchers. The author had widespread experience in transcribing dyadic conversation sessions; coding CTs and SRs; calculating inter-coder and intra-coder reliability; quantitatively analyzing contributions of CTs and SRs; and qualitatively profiling CTs and SRs in several previously published studies (e.g., [46,57,60]). These four research assistants (i.e., R1, R2, R3, and R4) were trained to transcribe video-recorded dyadic conversation sessions and code transcripts with CTs and SRs. Training sessions for transcribing conversation sessions and coding transcripts were adapted and modified from Guralnick and Paul-Brown [61] and Olswang, Svensson, Coggins, Beilinson, and Donaldson [62]. The transcription training sessions had a 5 min segmented dyadic conversation session, not included in the current study, between two HOAs viewed with the completed transcripts. Five randomly selected 5 min segmented dyadic conversation sessions were independently transcribed, and these transcripts were compared to the pre-completed transcripts. Reliability was established by dividing the number of agreements for occurrences by the number of agreements and disagreements of the occurrences and multiplying the quotient by 100 [63,64,65].

The occurrence of transcriptions (i.e., spoken words, gestures, unintelligible spoken words, and eye gazing directions) were only examined for reliability, and the exact duration of the pause time within and between the interlocutors was not examined due to not being the primary focus and not potentially affecting the current study. Competence was demonstrated when a minimum of 80% reliability on the transcriptions was reached for at least three of the segmented dyadic conversation sessions [61,64,66].

The coding training sessions first discussed the definitions of CTs and SRs. Second, these four research assistants independently coded two 5 min transcripts not collected in the current study and compared their coding with the pre-coded transcripts. The author and the research assistants discussed any discrepancies. Third, the research assistants independently coded CTs and SRs on two randomly selected 5 min portions of conversation transcripts, and these coded transcripts were compared to the pre-coded transcripts [57]. Reliability was established by dividing the number of agreements for occurrences by the number of agreements and disagreements of the occurrences and multiplying the quotient by 100 [63,64,65], and competence was demonstrated when a minimum of 80% reliability on these two coded transcripts was reached [61,64,66].

### 2.3. Research Settings and Materials

**Figure 1:**
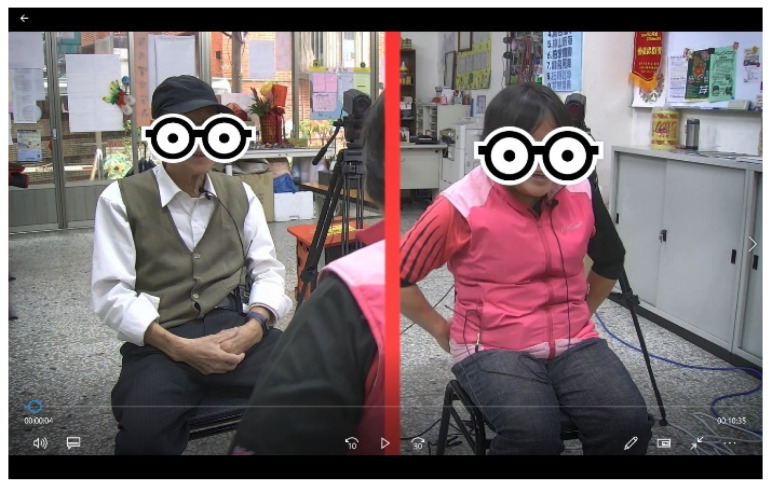
A Sample of Split-Screen Image.

Each core HOA casually conversed with their recommended HOAs in a quiet room where their daily conversation occurred, and these two HOAs were seated next to each other [57]. Two color video cameras (DATAVIDEO in Whittier, CA, USA, a registered trademark company: https://www.datavideo.com/ (accessed on 1 December 2022), BC-50 1080p HD Block Camera) were located behind these two HOAs, and each video camera captured a frontal view of the head and mid-torso separately. These two captured views were inputted into a special effects generator (DATAVIDEO in Whittier, CA, USA, HS-1300 6-Channel HD Portable Video Streaming Studio) to video-record a split-screen view, in which the HOA was on one side of the split screen and the other HOA on the other side (see Figure 1). The audio was recorded using two lapel microphones (SONY in Tokyo, Japan, a registered trademark company: https://www.sony.com.tw/zh (accessed on 1 December 2022), UWP-D21), and the microphones were clipped to the HOAs’ clothing at approximately the sternum level, and the audio recordings were sent to a sound mixer (YAMAHA in Shizuoka, Japan, a registered trademark company: https://tw.yamaha.com/index.html (accessed on 1 December 2022), MG10XU MIXER). The video cameras recorded a split-screen view, which was played back on the 17-inch color monitor (DATAVIDEO in Whittier, CA, USA, HS-1300 6-Channel HD Portable Video Streaming Studio), and the audio recordings were monitored through a pair of audio headsets (AUDIO-TECHNICA in Tokyo, Japan, a registered trademark company: https://www.audio-technica.com.tw/ (accessed on 1 December 2022), ATH-M30x). The integration of video recordings and audio recordings was then stored on a secure digital memory card (SANDISK in Milpitas, CA, USA, a registered trademark company: https://www.westerndigital.com/zh-tw/sandisk (accessed on 1 December 2022), Extreme SDXCⅠC10 U3 V30 64GB). The transcriptions were coded with CTs and SRs through Delve in New York, NY, USA (a registered trademark company: https://delvetool.com/ (accessed on 1 December 2022)), a software tool for analyzing qualitative data.

### 2.4. Procedures

Each dyad conversed once a week for five weeks, for a total of five dyadic conversation sessions per dyad and 50 dyadic conversation sessions. Sampling a greater amount of dyadic conversation sessions is needed to generalize validity [17]. Each dyadic conversation was video-recorded individually for at least but not limited to a 10 min conversation, which usually provides an appropriate representation of dyadic conversation [67], and the 10 min conversation has been used in several studies (e.g., [68,69,70]). After this time, the author signals the dyad to conclude the conversation. To make the conversation as natural as possible, no structured scripts were used [58,71,72]. The dyads were encouraged to engage in spontaneous and unstructured conversation [73], and conversational topics were chosen by the HOAs [72]. Additional conversational topics related to four aspects of daily living, including personal care (e.g., medication, dressing), mobility (e.g., using public transport, using taxis), housework (e.g., food shopping, cooking), and activities (e.g., reading a newspaper, seeing friends), were provided in occurrences of conversations lasting less than 10 min [74]. To diminish anxiety from the awareness of the video recording within the first 1–3 min after the video recording began, each HOA was told that the video recording is mainly focused on the conversation from the other HOA in their dyadic conversation [75]. The following instructions in Mandarin Chinese were given to each dyad before their conversation started [57]:

Your task today is to have a conversation with each other. Both of you will choose conversational topics. You might like to talk about daily living, including personal care, mobility, housework, and activities. No structured scripts will be used to keep the conversation as natural as possible. You will have about 10 min. I will signal you when 10 min has passed, so you may begin to wind up your conversation. If you feel you have finished your conversation before the end of 10 min, just let me know, and I will provide you with a list of possible topics to continue.

The mean length of each dyadic conversation was 10 min 7 s, ranging from 10 min 0 s to 11 min 0 s.

### 2.5. Data Analysis

The data analysis involved three steps: (a) transcribing dyadic conversation sessions; (b) coding CTs; and (c) coding SRs (i.e., AN, AU, and PR) [57]. The assistants were then paired as R1 with R2 and R3 with R4. For the analysis, first, all the video-recorded conversation sessions were transcribed through transcription notations adapted from the work of Tsai [56], as documented in Table S1. All spoken words, nonverbal conversation behaviors (e.g., head nod and eye gaze), and silences within and between utterances were transcribed precisely [62]. The first two research assistants (i.e., R1 and R3) transcribed the conversation sessions, and the other research assistants (i.e., R2 and R4) examined the accuracy of transcriptions before coding. Any discrepancies in the transcriptions were discussed to gain consensus [76], and any potential discrepancies were resolved [76,77].

Second, CTs were independently coded on an utterance-by-utterance basis by R1 and R2 [46]. A CT-A was coded to a CT contributed by the core HOAs, and CT-B was coded to a CT contributed by the recommended HOAs. Detailed operational definitions of CT coding adapted from the work of Tsai [56] and extracts illustrating different coding rules of CTs are documented in Table S2.

Third, three SRs (i.e., AN, AU, and PR) were independently coded on an utterance-by-utterance basis using Goffman’s [43] framework by R1 and R2. AN was defined as a person producing utterances (i.e., giving “voice” to the words) [43], and AU was defined as a person who selects words or infers meanings from incomplete spoken words, utterances (e.g., go), and/or nonverbal conversation behaviors (e.g., head nod and eye gaze) [43,44]. PR was defined as a person whose beliefs, positions, perspectives, personal information, and sentiments are established during the conversation [43,78]. An AN-A, AU-A, and PR-A were coded to an SR contributed to by the core HOAs, and an AN-B, AU-B, and PR-B were coded to an SR contributed to by the recommended HOAs. Detailed operational definitions of SR coding adapted from the work of Tsai, Scherz, and DiLollo [46] and extracted illustrating different coding rules of SRs are documented in Table S3.

### 2.6. Inter- and Intra-Coder Reliability

All 50 transcripts of the video-recorded conversation sessions from the ten dyads were coded by R1 and R2, with 25 transcripts for each. One transcript from each dyad, with ten transcripts in total, was randomly selected for calculating inter- and intra-coder reliability [57]. R1 and R2 coded the selected transcripts again one week after the initial coding, and R3 and R4 coded these selected transcripts without discussions allowed [62]. Point-by-point reliability was used to calculate inter- and intra-coder reliability in coding CTs and SRs [63]. Additional identical coding training sessions were provided before coding the transcripts of another dyad when a minimum of 80% reliability in each transcript was not reached [57]. The current study did not need additional coding training sessions. The mean inter-coder reliability was 99.30% (range = 98.66–100.00%) for CT coding and 97.25% (range = 93.42–99.01%) for SR coding. The mean intra-coder reliability was 98.84% (range = 96.90–100.00%) for CT coding and 96.75% (range = 94.78–100.00%) for SR coding.

### 2.7. Measures

The frequency of the coded CTs and SRs (i.e., animator, author, and principal) contributed by each HOA in each dyad and the number of each animator, author, and principal were tallied. Percentages of the coded CTs and SRs contributed by each HOA in each dyad were calculated to account for the diverse length of the dyadic conversation sessions [79]. The percentages of contributed CTs for each HOA were calculated by the number of CTs for each HOA divided by the total number of CTs of the dyad and multiplying the quotient by 100 [57]. The percentages of contributed SRs for each HOA were derived by dividing the number of SRs for each HOA by the total number of SRs of the dyad and multiplying the quotient by 100 [57]. A paired-sample *t*-test was conducted to compare the mean percentages of contributed CTs and SRs among these conversation dyads, similar to previous studies (e.g., [29,56,57]).

## 3. Results

### 3.1. Contributions of CTs

The mean percentages of the contributed CTs for each HOA in the dyads are documented in Table S4. A paired-sample *t*-test was calculated to compare the mean percentages of the CTs contributed by the core HOAs to the recommended HOAs among these 50 dyadic conversation sessions. The mean percentage of the core HOAs was 50.86 (*sd* = 6.40), and the mean percentage of the recommended HOAs was 49.14 (*sd* = 6.40). No significant difference (i.e., symmetry) between the core and recommended HOAs was found (*t*(49) = 0.948, *p* > 0.05).

### 3.2. Contributions of SRs

The mean percentages of the contributed SRs for each HOA in the dyads are documented in Table S5. Another paired-sample *t*-test was calculated to compare the mean percentages of the SRs contributed by the core HOAs to the recommended HOAs. The mean percentage of the core HOAs was 51.08 (*sd* = 6.95), and the mean percentage of the recommended HOAs was 48.92 (*sd* = 6.95). No significant difference (i.e., symmetry) between the core and recommended HOAs was found (*t*(49) = 1.099, *p* > 0.05).

## 4. Discussion

This study aimed to explore the quantitative contributions of CTs and SRs in Mandarin-Chinese-speaking conversation dyads between mutually familiar HOAs. Although typical aging processes might cause HOAs to have disturbances in speech (e.g., unintelligible speech), have declined language skills (e.g., declined comprehension of complex utterances and naming), and struggle to maintain conversations [12,20], the findings revealed quantitatively symmetrical contributions of CTs and SRs between HOAs.

### 4.1. Contributions of CTs

The study revealed that quantitatively symmetrical contributions of CTs occurred in Mandarin-Chinese-speaking conversation dyads between mutually familiar HOAs. The two HOAs sequentially took their obligatory CTs to co-construct their conversation process in their dyadic conversation, as Extract 2 illustrates below. From line 1 to 6, these two HOAs (i.e., A and B) had orderly and equal CTs; three CTs contributed by A and B each. Accordingly, quantitatively symmetrical contributions of CTs can be anticipated and demonstrate the occurrence of an ideal conversation, as proposed by Sacks, Schegloff, and Jefferson [28]. This may be because modesty influences Mandarin-Chinese-speaking conversation in Chinese culture, as argued by Wu [50].

Extract 2

1. A: 沒有辦法咬 (/méi yǒ ubàn fǎ jyué/, cannot chew)
2. B: 沒辦法咬就吃不下 (/méi bàn fǎ jyué jiòu chih sià/, can’t bite, can’t eat)
3. A: 用果汁機也不好吃 (/yòng guǒ jhih ji yě bù hǎo chih/, it’s not good to use a juicer)
4. B: 用果汁機可以吧 (/yòng guǒ jhih ji kě yǐ ba/, can I use a juicer?)
5. A: 那個營養差很多 (/nǎ ge˚yíng yang cha hěn duo/, that nutrition is much worse)
6. B: 喔營養差很多 (/wò yíng yang cha hěn duo/, oh, poor nutrition)

### 4.2. Contributions of SRs

The study revealed that quantitatively symmetrical contributions of SRs occurred in Mandarin-Chinese-speaking conversation dyads between mutually familiar HOAs. These mutually familiar HOAs who shared personal experience and world knowledge took different SRs to co-construct conversation content (i.e., conversation meanings) [12,23]. Extract 3 illustrates how each HOA (i.e., A and B) act as their own animator, author, and principal and as the author and principal for other HOAs. In lines 1, 2, 3, and 4, A and B act as their own animator, author, and principal. In line 5, B selects the correct name of the school (i.e., Taí Jhong Jia Shang) on A’s behalf, acting as their own animator and author but having A act as the principal. Consequently, A revises the correct name of the school (i.e., Taí Jhong Jia Shang) in line 6, and B interrupts A’s obligatory CT in line 8 5 s later. In line 10, B repeats A’s utterance in line 9, acting as their own animator, but A acts as the author and principal in line 10. A and B contributed 15 SRs each in Extract 13, and quantitatively symmetrical contributions of SRs can be anticipated.

Furthermore, several factors could explain these symmetrical contributions of SRs. First, although the current study did not investigate the frequencies of cooperative and intrusive interruptions, the other-oriented (e.g., loyalty and solidarity) sensitivity in Mandarin Chinese proposed by Li (2001) might influence the HOAs to cooperatively interrupt the other HOAs to continuously co-construct their conversation content as illustrated in line 8 in Extract 3. Second, the HOAs did have the other HOAs act as the author and principal but had themselves act as an animator when co-constructing their conversation content. The other-oriented sensitivity in Mandarin Chinese proposed by Li (2001) might again influence the HOAs to use discourse-building features (e.g., clear information units, cohesion, correct pronouns, and appropriate conjunctions), as reported by Lai [55], to alternate SRs appropriately (e.g., animator, author, and principal) and have the other HOAs act as the author and principal in continuingly co-constructing their conversation.

Extract 3

1. B: 那女生她現在讀 (/Nà Nyǔ Sheng Siàn Zaì Dú/, where does that girl study)
2. A: 高二了 (/Gao Èr Liaǒ/, already sophomore)
3. B: 高中二年級讀哪裡 (/Gao Jhòng Èr Nián Jí Dú Nǎ Lǐ/, where to study in the second year of high school)
4. A: 讀台中商職 (/Dú Taí Jhong Shang Jhíh/, study in Taichung Home Economics and Commercial High School)
5. B: 台中家商 (/Taí Jhong Jia Shang/, Taichung Home Economics and Commercial High School)
6. A: 台中家商 (/Taí Jhong Jia Shang/, Taichung Home Economics and Commercial High School)
7. B: 黑阿 (/Hei A/, yes)
8. B: (5.0) 坐公車去嗎 (/Zuò Gong Che Chyù Ma/, does take the bus)
9. A: 不一定 (/Bù Yí Dìng/, not certainly)
10. B: 不一定 (/Bù Yí Dìng/, not certainly)

### 4.3. Limitations and Future Research Directions

This study’s results should be interpreted with respect to three research limitations, which provide future research directions. First, although these HOAs all met the recruiting criteria, the validity of the findings might be influenced by diverse attributes (e.g., living styles, occupation, education levels, and sensitivity to the conversation) of the heterogeneous HOAs in Taiwan [57]. The effects of different attributes of HOAs on quantitative contributions of CTs and SRs in Mandarin-Chinese-speaking conversation dyads should be investigated. Second, these dyadic conversation sessions were video-recorded in natural conversational contexts where these HOAs had daily conversations. In the current study, these HOAs’ natural conversation contexts might not fully represent all everyday conversation contexts (e.g., home, supermarkets, and stations) [20]. The effects of different conversation contexts on the quantitative contributions of CTs and SRs in Mandarin-Chinese-speaking conversation dyads should be investigated. Third, the modes of conversation behaviors (e.g., verbal and nonverbal conversation behaviors) that occurred among these HOAs were not explored in the current study. Although the conversations focused on co-constructions of processes and content through CTs and SRs rather than modes of conversation behaviors [56], quantitative contributions of different modes of conversation behaviors from the HOAs should be addressed to better understand their dyadic conversation in their natural conversation contexts.

### 4.4. Implications

There are several practical implications from the findings of the current study. First, normative information on the quantitative contributions of CTs and SRs in Mandarin-Chinese-speaking conversation dyads between mutually familiar HOAs can be acknowledged by the community care centers supervised by the MOHW [7] in Taiwan. HOAs conversing daily with other HOAs in community care centers might have a more supportive environment to connect to the surrounding community (i.e., caregivers and neighbors) and improve friendships [10,11,12,13,14]. Second, in the common phenomenon of young-old adults caring for old-old adults in Taiwan, quantitatively symmetrical contributions of CTs and SRs were not influenced by typical aging processes. Misperceptions about suffering from neurological disease (e.g., stroke, dementia) among HOAs might be reduced [17]. These Mandarin-Chinese-speaking, mutually familiar, healthy young-old adults and old-old adults who have shared personal experience and shared world knowledge in Taiwan can play important roles for each other in co-constructing their dyadic conversation.

## 5. Conclusions

This study explored the quantitative contributions of CTs and SRs in Mandarin-Chinese-speaking conversation dyads between mutually familiar HOAs. Quantitatively symmetrical contributions of CTs and SRs occurred in these Mandarin-Chinese-speaking dyadic conversations. Modesty, proposed by Wu [50], and other-oriented (e.g., loyalty and solidarity) sensitivity, proposed by Li (2001), play critical roles in co-constructing the conversation process (e.g., completing incomplete conversation turns) and content (e.g., building mutual meanings) in Chinese-speaking dyadic conversation. Although typical aging processes might change conversations (e.g., unintelligible speech) [17,18,19,20], both Mandarin-Chinese-speaking HOAs serve as active interlocutors for CTs and SRs to co-construct their conversation process and content in their dyadic conversations.

## Figures and Tables

**Table 1:** Demographic Characteristics of 10 Core HOAs.

#	Gender	Age	Years of Schooling	Handedness	Most Spoken Language	Occupation	Hearing Aids	Glasses
1	Male	76	16	Right	Mandarin Chinese	Retired	N/A	N/A
2	Male	83	6	Right	Mandarin Chinese	Retired	N/A	Yes
3	Male	71	6	Right	Mandarin Chinese	Retired	N/A	N/A
4	Male	85	12	Right	Mandarin Chinese	Retired	N/A	Yes
5	Male	76	12	Right	Mandarin Chinese	Retired	N/A	N/A
6	Male	76	12	Right	Mandarin Chinese	Retired	N/A	N/A
7	Male	72	6	Right	Mandarin Chinese	Retired	N/A	N/A
8	Male	73	6	Right	Mandarin Chinese	Retired	N/A	N/A
9	Male	84	18	Right	Mandarin Chinese	Retired	N/A	N/A
10	Male	89	12	Right	Mandarin Chinese	Retired	N/A	Yes

**Table 2:** Demographic Characteristics of 10 Recommended HOAs.

#	Gender	Age	Years of Schooling	Handedness	Most Spoken Language	Relationship of the Reference	Hearing Aids	Glasses
1	Female	81	6	Right	Mandarin Chinese	Friend	N/A	Yes
2	Female	68	12	Right	Mandarin Chinese	Friend	N/A	Yes
3	Female	70	9	Right	Mandarin Chinese	Couple	N/A	N/A
4	Female	84	6	Right	Mandarin Chinese	Couple	N/A	Yes
5	Female	79	6	Right	Mandarin Chinese	Friend	N/A	N/A
6	Female	69	9	Right	Mandarin Chinese	Couple	N/A	N/A
7	Female	70	6	Right	Mandarin Chinese	Couple	N/A	N/A
8	Female	71	6	Right	Mandarin Chinese	Couple	N/A	N/A
9	Female	80	12	Right	Mandarin Chinese	Couple	N/A	Yes
10	Female	85	9	Right	Mandarin Chinese	Couple	N/A	Yes

## Data Availability

The data presented in this study are available in Tables S1 and S2, and Supplementary Materials.

## Supplementary Materials

The following supporting information can be downloaded at: https://www.mdpi.com/article/10.3390/bs13020134/s1, Table S1: Transcription Notations; Table S2: Definitions of CTs Coding Rules and Extracts; Table S3: Definitions of SRs Coding Rules and Extracts; Table S4: Quantitative Contributions of CTs in Mandarin Chinese-Speaking HOAs Conversation Dyads; Table S5: Quantitative Contributions of SRs in Mandarin Chinese-Speaking HOAs Conversation Dyads.
