# Supplementary material for: Dyadic Conversation between Mandarin-Chinese-Speaking Healthy Older Adults: From Analyses of Conversation Turns and Speaking Roles
Source: Behav Sci (Basel). 2023 Feb 5;13(2):134. doi: 10.3390/bs13020134 (PMC9952709; doi:10.3390/bs13020134)
Supplement: Supplementary file 1 [file behavsci-13-00134-s001.zip › behavsci-2121611-supplementary.pdf]

Supplemental Materials

Table S1

Transcription Notations

Notation	Description	Example
Words	Transcribe spoken words	1 A: 蘋果 (/píng guǒ/, apple)
	Link simultaneous spoken words and nonverbal conversation behaviors together in one conversation line	1 A: 沒問題 (/mò wùn tí/, no problem)?<br>((head nod))
	Mark continuing spoken words and nonverbal conversation behaviors that are continued and discontinued by another (i.e., overlapping)	1 A: 蘋果 (/píng guǒ/, apple)<br>B: 蘋果 (/píng guǒ/, apple)
(5.0)	Indicate number in parentheses, presented in seconds within and between utterances	1 A: 買蘋果 (/mǎi píng guǒ/, buy apples)<br>2 A: (5.0)沒問題 (/méi wèntí/, not problem)
(( ))	Specify nonverbal conversation behaviors (e.g., head shake) in a double parenthesis	1 A: ((head nod))
(Sounds)	Present sounds that are hard to transcribe into single parentheses, and the empty single parentheses mean utterance cannot be heard.	1 A: 蘋果 (/píng guǒ/, apple)<br>2 B: (/n/)
.____.	Indicate a gaze on an item in a line with two dots	1 A: 蘋果 (/píng guǒ/, apple)<br>.蘋果 (apple).

Note. Adapted from *The effect of familiarity of conversation partners on conversation turns contributed by augmented and typical speakers*, by M.-J. Tsai, 2013, *Research in Developmental Disabilities, 34*(8), 2326-2335.

Table S2

Definitions of CTs Coding Rules and Extracts

Coding rules		Extracts	
Rule 1	Code a conversation turn when the speaking interlocutor changes between the speaking and listening interlocutors.	1	A: 蘋果 (/píng guǒ/, apple) *[CT-A]*
		2	B: ((head nod)) *[CT-B]*
Rule 2	Code a conversation turn when an overlapping spoken words (e.g., "yes") and nonverbal conversation behaviors (e.g., head nod) occurs from the listening interlocutor in order to answer the speaking interlocutor's question while the speaking interlocutor is still speaking.	1	A: 蘋果 (/píng guǒ/, apple) *[CT-A]*<br>B: 蘋果 (/píng guǒ/, apple) *[CT-B]*
Rule 3	Code two conversation turns for the speaking interlocutor. The speaking interlocutor initiates a conversation (e.g., asking a question, giving a greater than 2-s pause, gazing at the listening interlocutor) in order to yield a conversation floor to the listening interlocutor, but no response from the listening interlocutor occurs, and then the speaking interlocutor begins another conversation.	1	A: 蘋果 (/píng guǒ/, apple) *[CT-A]*
		2	A: (5.0) 沒問題 (/méi wèntí/, not problem) *[CT-A]*
Rule 4	Code a conversation turn when the speaking interlocutor has no pause(s) in between conversation.	1	A: 蘋果，裝那個袋子 (/píng guǒ jhuang nǎ ge˚dài zih˚/, place apples in that bag) *[CT-A]*
Rule 5	Do not code a conversation turn when overlapping spoken words (e.g., "yes") or nonverbal conversation behaviors (e.g., head nod) from the listening interlocutor while the speaking interlocutor is speaking are used to validate attention to the speaking interlocutor without contributing conversation meanings.	1	A: 裝袋子 (/jhuang dài zih˚/, place in bag) [CT-A]<br>B: ((head nod))

Note. Adapted from *The effect of familiarity of conversation partners on conversation turns contributed by augmented and typical speakers*, by M.-J. Tsai, 2013, *Research in Developmental Disabilities, 34*(8), 2326-2335.
[CT-A] = A conversation turn was contributed by A.
[CT-B] = A conversation turn was contributed by B.

Table S3

Definitions of SRs Coding Rules and Extracts

Coding rules		Extracts
Rule 1	Code *Animator* when the speaking interlocutor produces spoken words (e.g., “yes”)	1 A: 蘋果 (/píng guǒ/, apple) *[AN-A]*<br>2 B: ((head nod))
Rule 2	Code *Author* when the speaking interlocutor selects or infers the words or meanings for conversation behaviors	1 A: 蘋果 (/píng guǒ/, apple) *[AU-A]*<br>2 B: ((head nod)). *[AU-B]*
Rule 3	Code *Principal* when the person whose beliefs, positions, perspectives, personal information, and sentiments are established in the speaking interlocutor’s conversation behaviors	1 A: 蘋果 (/píng guǒ/, apple) *[PR-A]*<br>2 B: ((head nod)) *[PR-B]*
Rule 4	Code once when the speaking interlocutor produces multiple conversation behaviors that do not specifically relate to previous conversation behaviors	1 B: 那裡 (/nali/, there)<br>2 A: 蘋果裝那個袋子 (/píng guǒ jhuang nǎ ge˚dài zih˚/, place apples in that bag) *[AN-A]; [AU-A]; [PR-A]*
Rule 5	Code each segment of the conversation behaviors when the speaking interlocutor repeats a previous conversation behavior and then continues additional conversation behaviors with new information.	1 A: 蘋果 (/píng guǒ/, apple)<br>2 B: 蘋果 (/píng guǒ/, apple) *[AN-B];[AU-A]; [PR-A]* 沒問題 (/méi wèntí/, not problem). *[AN-B]; [AU-B]; [PR-B]*

Rule 6	Do not code *Animator* for nonverbal conversation behaviors (e.g., head nod, head shake)	1	B: 蘋果 (/píng guǒ/, apple) *[AN-B]; [AU-B]; [PR-B]*
		2	A: ((head nod)) *[AU-B]; [PR-A]*
Rule 7	Do not code when a conversation turn is not coded	1	A: 蘋果 (/píng guǒ/, apple) *[AN-A]; [AU-A]; [PR-A]*
		2	B: 蘋果 (/píng guǒ/, apple) *[AN-B]; [AU-B];[PR-B]*<br>A: ((head nod))

Note. Adapted from *Conversation of augmented and typical speakers—Speaking roles versus conversation turns, by Tsai, M.-J., Scherz, J., & DiLollo, A. 2011, Asia Pacific Journal of Speech, Language and Hearing, 14(4), 179-185.*

[AN-A] = Animator was contributed by A.

[AN-B] = Animator was contributed by B.

[AU-A] = Author was contributed by A.

[AU-B] = Author was contributed by B.

[PR-A] = Principle was contributed by A.

[PR-B] = Principle was contributed by B.

Table S4

Quantitative Contributions of CTs in Mandarin Chinese-Speaking HOAs Conversation Dyads

Dyadic conversation	Contributed CT-A		Contributed CT-B	
	n	%	*n*	%
1	66	49.25%	68	50.75%
2	31	47.69%	34	53.13%
3	67	50.00%	67	50.00%
4	26	43.33%	34	56.67%
5	35	50.00%	35	49.30%
6	47	50.54%	46	48.94%
7	22	50.00%	22	51.16%
8	39	48.75%	41	51.25%
9	27	51.92%	25	48.08%
10	46	52.27%	42	47.19%
11	22	43.14%	29	56.86%
12	17	43.59%	22	56.41%
13	12	46.15%	14	53.85%
14	9	32.14%	19	67.86%
15	13	46.43%	15	53.57%
16	15	51.72%	14	48.28%
17	13	65.00%	7	35.00%
18	34	45.95%	40	54.05%
19	19	55.88%	15	44.12%
20	17	45.95%	20	54.05%
21	42	46.15%	49	53.85%
22	51	51.52%	48	48.48%
23	32	53.33%	28	46.67%
24	22	53.66%	19	46.34%
25	18	47.37%	20	52.63%
26	103	51.24%	98	48.76%
27	73	51.41%	69	48.59%
28	77	50.00%	77	49.68%
29	79	50.32%	78	49.68%
30	68	50.37%	67	49.63%
31	51	66.23%	26	33.77%
32	24	64.86%	13	35.14%

33	22	59.46%	15	40.54%
34	52	60.47%	34	39.53%
35	31	50.82%	30	49.18%
36	74	56.06%	58	43.94%
37	70	52.24%	64	47.76%
38	74	51.75%	69	48.25%
39	63	49.61%	64	50.39%
40	67	50.76%	65	48.87%
41	62	50.82%	60	49.18%
42	44	58.67%	31	41.33%
43	27	50.00%	27	50.00%
44	38	60.32%	25	39.68%
45	70	53.44%	61	46.21%
46	34	47.89%	37	52.11%
47	17	37.78%	28	62.22%
48	18	56.25%	14	43.75%
49	37	49.33%	38	51.35%
50	16	41.03%	23	58.97%

Note. CT-A and CT-B reflect a CT contributed by the core HOAs and the recommended HOAs, respectively.

Table S5

Quantitative Contributions of SRs in Mandarin Chinese-Speaking HOAs Conversation Dyads

#	AN-A	AU-A	PR-A	Contributed SR-A	AN-B	AU-B	PR-B	Contributed SR-B
1	*n*	*n*	*n*	%	*n*	*n*	*n*	%
2	66	74	75	52.83%	68	60	64	47.17%
3	30	29	35	48.45%	34	36	30	51.55%
4	67	67	73	51.49%	67	68	60	48.51%
5	26	26	28	45.20%	34	34	29	54.80%
6	36	34	28	46.67%	34	37	41	53.33%
7	48	46	43	49.28%	46	47	48	50.72%
8	21	17	13	38.64%	22	28	31	61.36%
9	39	40	42	50.63%	41	40	37	49.37%
10	27	24	23	47.44%	25	28	29	52.56%
11	47	53	53	57.95%	41	35	35	42.05%
12	22	23	29	48.37%	29	28	22	51.63%
13	17	16	19	44.07%	22	23	21	55.93%
14	12	12	13	47.44%	14	14	13	52.56%
15	9	10	14	39.29%	19	18	14	60.71%
16	13	14	15	49.41%	15	15	13	50.59%
17	15	15	15	51.72%	14	14	14	48.28%
18	13	13	17	71.67%	7	7	3	28.33%
19	34	38	37	48.66%	40	36	39	51.34%
20	19	19	21	57.84%	15	15	13	42.16%
21	17	16	16	44.14%	20	21	21	55.86%
22	42	45	45	48.89%	47	46	45	51.11%
23	51	52	57	54.24%	47	47	41	45.76%
24	32	31	38	56.11%	28	29	22	43.89%
25	22	20	22	52.03%	19	21	19	47.97%
26	18	18	20	49.56%	19	20	18	50.44%
27	103	88	82	45.27%	99	113	118	54.73%
28	73	62	59	45.54%	69	80	83	54.46%
29	78	62	61	43.79%	76	91	91	56.21%
30	79	76	75	48.94%	78	81	81	51.06%
31	68	66	68	49.75%	67	69	68	50.25%
32	51	53	53	67.97%	26	24	24	32.03%

33	24	24	24	64.86%	13	13	13	35.14%
34	22	23	24	62.73%	15	13	13	37.27%
35	52	52	59	62.93%	34	35	27	37.07%
36	31	31	36	53.55%	30	30	25	46.45%
37	74	75	59	52.13%	58	57	76	47.87%
38	70	69	65	51.13%	64	65	66	48.87%
39	74	75	46	45.35%	69	68	98	54.65%
40	63	66	56	48.94%	64	61	68	51.06%
41	68	63	53	46.70%	64	69	77	53.30%
42	62	56	73	51.90%	60	66	51	48.10%
43	44	42	49	60.00%	31	33	26	40.00%
44	27	26	32	52.80%	27	28	21	47.20%
45	38	35	30	54.50%	25	28	33	45.50%
46	71	67	77	55.27%	60	64	50	44.73%
47	34	34	34	47.44%	37	37	39	52.56%
48	17	18	22	42.22%	28	27	23	57.78%
49	18	18	20	58.33%	14	14	12	41.67%
50	36	37	38	48.90%	38	38	40	51.10%
51	16	17	15	41.03%	23	22	24	58.97%

Note. SR-A and SR-B reflect a SR contributed by the core HOAs and the recommended HOAs, respectively.
